# Acquired Localized Hypertrichosis Induced by Rivastigmine

**DOI:** 10.1155/2016/7296572

**Published:** 2016-03-17

**Authors:** Adrian Imbernón-Moya, Sebastian Podlipnik, Fernando Burgos, Elena Vargas-Laguna, Antonio Aguilar-Martínez, Eva Fernández-Cogolludo, Miguel Angel Gallego-Valdes

**Affiliations:** ^1^Department of Dermatology, Hospital Universitario Severo Ochoa, Avenida de Orellana, Leganés, 28911 Madrid, Spain; ^2^Department of Dermatology, Hospital Clinic, Carrer de Villarroel 170, 08036 Barcelona, Spain; ^3^Department of Pathology, Hospital Universitario Severo Ochoa, Avenida de Orellana, Leganés, 28911 Madrid, Spain

## Abstract

Hypertrichosis is the excessive hair growth in any area of the skin surface. Acquired localized hypertrichosis may be secondary to multiple causes and there is a secondary form due to several drugs, which is usually reversible with discontinuation of the causative agent. Rivastigmine is a reversible and competitive inhibitor of acetylcholinesterase and butyrylcholinesterase used for symptomatic treatment of Alzheimer dementia and Parkinson's disease. It has an adequate safety profile and cutaneous side effects are unusual. Irritant contact dermatitis, allergic dermatitis, baboon syndrome, and cutaneous rash due to rivastigmine have been reported. We report on a Caucasian 80-year-old male with personal history of Alzheimer's disease. The patient started therapy with oral rivastigmine one month prior to clinical presentation of localized hypertrichosis on both forearms. Norgalanthamine has been shown to promote hair growth activity via the proliferation of dermal papilla. Acetylcholinesterase inhibitors can induce hair growth.

## 1. Introduction

Irritant contact dermatitis, allergic dermatitis, baboon syndrome, and cutaneous rash due to rivastigmine have been reported. However, to our knowledge, no description of rivastigmine producing hypertrichosis is reported in the literature to date. We report a case of drug-induced acquired localized hypertrichosis associated with oral rivastigmine use.

## 2. Case Presentation

We report the case of a Caucasian 80-year-old male referred to the Dermatology Department due to progressive asymptomatic hair growth on both forearms for three months. He had a personal history of chronic obstructive pulmonary disease and Alzheimer's disease with severe cognitive impairment, with no family or dermatological history. The patient denied sun exposure, infection, trauma, bites, or contact with chemicals, but he started taking oral rivastigmine (3 mg every 12 hours) one month prior to clinical presentation of the hypertrichosis. The patient denied taking other drugs.

**Figure 1:**
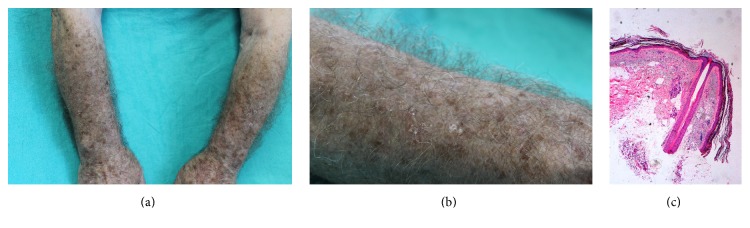
(a) Increased density of pigmented and thickened terminal hair in the forearms. (b) Increased density of pigmented and thickened terminal hair in the forearms. (c) Terminal medullated and pigmented hair follicle in the dermis without apparent alteration (hematoxylin and eosin, ×4).

Dermatological examination showed increased density of pigmented and thickened terminal hair and several actinic keratoses on his dorsal sides of forearms with symmetrical distribution (Figures 1(a) and 1(b)). There were no other cutaneous findings and general physical examination was normal.

All the following laboratory evaluations were in the normal range: biochemical parameters, complete blood cell count, hemostasis albumin, serum protein electrophoresis, thyroid-stimulating hormone (TSH), testosterone, dihydrotestosterone, dehydroepiandrosterone sulphate (DHEAS), androstenedione, cortisol, L-lactate dehydrogenase (LDH), beta-2-microglobulin, and tumor markers (PSA, CEA, CA 19.9, and CA 125). Syphilis serology, HCV, HBV, and HIV were negative. The chest radiograph and abdominal ultrasound did not show any pathological findings.

Microscopic analysis of a hair sample did not show any abnormalities in its structure. Histologic examination of the hair follicles obtained from a punch biopsy demonstrated that the hairs were of terminal type since they were medullated and pigmented and penetrated deep into the dermis. No apparent alteration was observed (Figure 1(c)).

Due to normal diagnostic tests and skin biopsy, we arrived at the diagnosis of acquired localized hypertrichosis secondary to the use of rivastigmine. Given the benign nature of the entity and the absence of symptoms, it was decided to conduct a clinical follow-up, without stopping treatment. The patient has been stable in clinical visits during a period of one year, without presenting extension of the hypertrichosis.

## 3. Discussion 

Hypertrichosis is the excessive hair growth in any area of the skin surface, whereas hirsutism is a distinct entity characterized by the appearance of hair in children and/or women, with an adult male pattern distribution associated with hormonal changes. Hypertrichosis is classified as congenital or acquired, and there are localized and generalized forms. Moreover, hair in hypertrichosis is usually longer than expected and may consist of any hair type (lanugo, vellus, or terminal) [1].

Acquired localized hypertrichosis may be secondary to multiple causes including Becker's nevus, chemicals, neoplasms, bone fractures, use of casts and splints, trauma, friction, venous malformations, thrombosis, osteomyelitis, HIV, systemic lupus erythematosus, and linear scleroderma [1, 2]. Moreover, there is a secondary form due to drugs, which is usually reversible with discontinuation of the causative agent, and it has been associated with numerous treatments (Table 2) [1–3].

Rivastigmine is a reversible and competitive inhibitor of acetylcholinesterase and butyrylcholinesterase used for symptomatic treatment of Alzheimer dementia and Parkinson's disease. It can be administered orally or transdermally and it has an adequate safety profile [4]. Side effects are dose dependent and occur in eight to nine percent of patients. Main side effects are gastrointestinal (anorexia, dyspepsia, nausea, vomiting, diarrhea, and abdominal pain) which are usually mild and transient, followed by neurological disorders (asthenia, headache, drowsiness, confusion, anxiety, and agitation) and cardiovascular problems (hypertension and orthostatic hypotension) [4, 5].

Cutaneous side effects are rare, with a frequency of 7% in users of transdermal patches, which are generally mild. However, in approximately 2.5% of the patients, it is necessary to interrupt the medication due to severe skin reactions. Irritant contact dermatitis is the most common cutaneous side effect of rivastigmine patch users. It manifests itself with geometric eczematous lesions, which are located in the area where the transdermal patch was applied. Signs and symptoms of irritant contact dermatitis may be minimized by rotation of the application site, careful removal of the patch, and application of the patch to clean, dry, nonhairy area of healthy skin [4–7].

In addition, there have been some case reports of allergic dermatitis and baboon syndrome (Table 1) [4–11]. These cases have developed a cutaneous rash, without systemic symptoms or laboratory abnormalities. The evolution is self-limited with discontinuation of the drug and sometimes oral or topical corticosteroids associated with oral antihistamines are necessary. Moreover, there have been reports of cross-reactivity with other acetylcholinesterase inhibitors such as galantamine, which have triggered identical skin symptoms [8].

Acetylcholinesterase inhibitors can induce hair growth. Norgalanthamine, a principal extract of the plant* Crinum asiaticum*, has been shown to promote hair growth activity via the proliferation of dermal papilla [12]. We attribute the cause of hypertrichosis to rivastigmine by the temporal relationship, without finding another possible cause. The patient has not taken any other drugs. He has not been given topical chemicals and the analytical and imaging tests were normal. Rivastigmine was not stopped because it was necessary for the underlying disease, so we could not check the resolution of hypertrichosis.

## Figures and Tables

**Table 1:** Reported cases of adverse skin effects by rivastigmine.

	Gender	Age (years)	Route of administration	Latency time until the rash	Diagnosis	Evolution
Golüke et al. [8]	Male	74	Transdermal patch (9.5 mg/day)	1 year	Localized allergic contact dermatitis	Drug discontinuation Resolution in days Recurrence with galantamine

Makris et al. [9]	Female	85	Transdermal patch (9.5 mg/day) Oral (6 mg/day)	3 weeks	Disseminated allergic contact dermatitis	Drug discontinuation Resolution in 2 weeks

Allain-Veyrac et al. [10]	Male	88	Oral (3 mg/day)	3 weeks	Baboon syndrome	Drug discontinuation Resolution in 2 weeks

Greenspoon et al. [6]	Female	65	Transdermal patch (Dose NA)	Several weeks	Disseminated allergic contact dermatitis	Drug discontinuation Resolution in several weeks

Grieco et al. [7]	Male	75	Transdermal patch (9.5 mg/day)	2 weeks	Disseminated allergic contact dermatitis	Drug discontinuation Resolution time NA

Monastero et al. [11]	Female	73	Oral (3 mg/day)	5 days	Baboon syndrome	Drug discontinuation Resolution in 2 weeks

NA: not available.

**Table 2:** Reported cases of acquired hypertrichosis due to drugs.

Widespread involvement (^*∗*^)	Cyclosporin A Corticosteroids Interferon alpha Penicillin Streptomycin Phenytoin Diphenylhydantoin Spironolactone Zidovudine Acetazolamide Latanoprost Bimatoprost Psoralens Diazoxide Minoxidil

Localized involvement	Topical latanoprost Topical bimatoprost Topical minoxidil

^*∗*^Usually generalized involvement with scalp, frontal region, trunk, and extremity involvement.
